# Peri-Abortion Contraceptive Choices of Migrant Chinese Women: A Retrospective Review of Medical Records

**DOI:** 10.1371/journal.pone.0040103

**Published:** 2012-06-29

**Authors:** Sally B. Rose, Zhang Wei, Annette J. Cooper, Beverley A. Lawton

**Affiliations:** Department of Primary Health Care and General Practice, University of Otago, Wellington, New Zealand; Indiana University, United States of America

## Abstract

**Background:**

Migrant Asian women reportedly have low levels of contraceptive use and high rates of abortion in New Zealand. Chinese make up the largest proportion of migrant Asian in New Zealand. This study aimed to describe the contraceptive choices of Chinese women seeking abortion; to examine method choice in relation to demographic characteristics (including length of stay) and to determine whether Chinese women were over-represented among abortion clinic attendees.

**Methods:**

Retrospective review of medical records at a public hospital abortion clinic involving 305 Chinese women. Previously collected data for European (n = 277) and Maori women (n = 128) were used for comparative analyses. Regression analyses explored correlates of contraceptive method choice. Population census data were used to calculate rates of clinic attendance across ethnic groups.

**Results:**

Chinese women were not over-represented among clinic attendees, and had similar rates of contraceptive non-use pre-abortion as women in comparison groups. Use of the oral contraceptive pill by Chinese was lower pre-abortion than for other ethnic groups, but choice of this method post-abortion was similar for Chinese (46.9%, 95% CI 41–52.7) and European women (43.7%, 95% CI 37.8–49.7). Post-abortion choice of an intrauterine device did not differ significantly between Chinese (28.9%, 95% CI 23.8–34.3) and Maori women (37%, 95% CI 28.4–45.7), but was higher than uptake of this method by European women (21.7%, 95% CI 17–27.0). Age, parity and previous abortion were significant predictors of post-abortion method choice by Chinese women (p<0.05).

**Conclusions:**

Following contraceptive counseling at the clinic, Chinese women chose more effective contraceptive methods for use post-abortion than they had used previously. As the population of migrant Chinese in New Zealand continues to increase, strategies are urgently needed to provide new arrivals with appropriate information and advice about contraception and where to access it, so women can be better prepared to avoid unplanned pregnancy.

## Introduction

The sexual reproductive health of Asian women in New Zealand has received negative attention in the media and in public health circles over the past decade. [1] Chinese are the largest ethnic group within the fast-growing Asian population in New Zealand, [2] and the majority (78%) are overseas-born. [3] Concerns arose following a report in 2001 that revealed Asian women had the highest abortion ratio of all ethnic groups in New Zealand, at 311 abortions per 1000 pregnancies. [4] By comparison, European women had 210 abortions per 1000 and Maori (the indigenous people of New Zealand) women had 257 per 1000 pregnancies. [4] The 2001 report also highlighted the higher rate of terminated pregnancies among teenage Asian women compared to women in other ethnic groups. [4] In 2003, a study on the use of contraception by Asian women (most of whom were Chinese migrants on student visas) seeking abortion at a private New Zealand clinic showed that 80% of the women had been using no form of contraception prior to becoming pregnant. [5] It was suggested in several published papers and the New Zealand media that immigrant Chinese women were using abortion as a form of contraception, [1] [5] a perception that has been reiterated again more recently. [6] These issues and the related discourse on the ‘problem’ of Asian women's sexuality were critically discussed by Simon-Kumar in 2009. [1]

Prevention of unplanned pregnancies requires consistent use of effective contraception. Women using methods that do not rely on user compliance for efficacy (particularly intrauterine and implantable contraceptives) are less likely to face unwanted pregnancy. [7] Research in New Zealand and elsewhere has suggested that Chinese women favour barrier or ‘natural’ (fertility awareness, rhythm, periodic abstinence) methods of contraception over hormonal preparations, and that the oral contraceptive pill (OCP) is perceived by Chinese women as harmful. [5], [8] Fertility awareness based methods are known to be less reliable than hormone-containing methods, [9] and concerns have been raised about whether women have adequate knowledge of such methods to use them correctly and consistently in order to avoid pregnancy. [10] Together these factors may be putting Chinese women at greater risk for unplanned pregnancy.

Women presenting for abortion in New Zealand receive pre-abortion counseling, and discussion about pregnancy prevention and contraception is a key component. Past use of contraception is discussed and information provided about methods available for use post-abortion, with discussion of risks, benefits and side effects. Women are encouraged to have a contraceptive plan in place when discharged following the abortion. Four methods of contraception can be initiated prior to discharge following an abortion. Immediate insertion of an intrauterine device (IUD) is available to women presenting for first trimester surgical abortion, and eligibility is determined on a case-by-case basis for women presenting for second trimester abortion. The copper multiload IUD is subsidized by the government so is free of charge, while the Mirena LNG-IUS costs approximately NZ$300. Women choosing depot medroxyprogesterone acetate (DMPA) receive a (free) injection after the abortion, prior to discharge from the clinic. Contraceptive implants are now subsidized (so are also free) but were not available during the course of the present study. Prescriptions are written for the OCP that incur a small charge to patients when they are filled.

In light of the reports on contraceptive non-use and the higher abortion ratios, we aimed to describe the peri-abortion contraceptive choices of Chinese women presenting to a public hospital abortion clinic in New Zealand. We compared method choice across three ethnic groups (Chinese, European and Maori) and as secondary aims, we sought to examine correlates of contraceptive method choice including length of stay in New Zealand. Finally, we used population census data to determine whether Chinese women were presenting for abortion at disproportionately higher rates per population than women in other ethnic groups.

## Materials and Methods

### Setting

This study was conducted in 2010 at the Wellington public hospital abortion clinic where the second highest number of abortions are performed annually in New Zealand. This clinic is the sole service provider in the area (so data collection included all women seeking abortion in this region) and provides a free service to New Zealand residents. Non-residents are charged up to NZ$1800 for an abortion. The service provided by this clinic requires a referral by a primary-care provider (family physician) and offers a choice of surgical or medical abortion. Patient-centered contraceptive counseling is provided by the clinic team (social workers, doctors, nurses) prior to the procedure. The majority of Chinese women presenting to this clinic are seen by a Chinese counselor (who is fluent in Cantonese), and interpreting services are provided if required.

Ethical approval was granted for this study by the Central Regional Ethics Committee, Wellington, New Zealand on 14th December 2009 (Ref CEN 09/11/082). Written consent was not given by the patients for their information to be stored in the hospital database and used for research. Collection of health information without consent is permitted under section 6.41 of the New Zealand Ethical Guidelines for Observational Studies. [11]

### Study Design

The study was powered for the primary outcome: post-abortion method choice by ethnic groups. A sample size of at least 300 Chinese women was deemed to be sufficient to detect as statistically significant differences in post-abortion method choices by women across three comparison groups. Starting with the most recent data (February 2010), data were retrospectively collected from the medical records of Chinese women identified from an extract of electronically collected hospital clinic data. We collected National Health Index (NHI, a uniquely assigned hospital number), date of procedure, date of birth, self-identified patient ethnicity, obstetric history and procedure details. Archived medical records were requested from the hospital patient records department for these women to obtain data that are not captured electronically including: country of birth, years of residence in New Zealand (‘length of stay’), visa status, contraceptive method in use pre-abortion, and method chosen for use at discharge. Socioeconomic deprivation was estimated using the New Zealand Deprivation index which is an area-based measure of socio-economic deprivation based on individuals residential address where scores range from 1 (least deprived) to 10 (most deprived). [12] To compare contraceptive choices made by women of other ethnicities, we used data that were collected in a review of patient records as part of an earlier study at the same clinic that involved 277 European and 128 Maori women (ethnic groups that make up 75% of women presenting for abortion at this clinic). The earlier data collection took place between June and August 2008 to provide a baseline measure of contraceptive use prior to an intervention study that aimed to increase post-abortion use of long-acting reversible contraceptive methods (LARC). [13]

As secondary outcomes we sought to explore correlates of post-abortion method choice including demographic and clinical characteristics and length of stay (using year of arrival to calculate time in New Zealand). Regional population census data (2006) were used to calculate percentages of reproductive-aged (15–44 years) women presenting for abortion by ethnic group. [3] [14] The ethnic group ‘Asian’ as described in this paper uses the New Zealand Census definition that is based on an individual's own self-identification with a particular ethnic group or groups. [15]

### Data analysis

Demographic and clinical data were tabulated for comparison across the three ethnic groups. Chi-square tests were used to test for overall differences between demographic and clinical characteristics by ethnic group. Pairwise comparisons were used to determine where any overall differences lay. Contraception data (methods used pre- and post-abortion) were collated and 95% confidence intervals calculated to compare method choice across groups.

Multivariate logistic regression analyses were used to examine predictors of contraceptive method choice by Chinese women post-abortion including demographic (age, socioeconomic deprivation and time in New Zealand) and clinical co-variates (pregnancy history). A generalized logit regression approach was used to allow for comparison between three categories of post-abortion contraceptive methods (as compared to binary logistic regression, which only allows for two outcome levels.) Three categories of contraceptive method were defined for the regression outcome: long-acting reversible contraceptive (LARC) methods that included IUDs and DMPA, OCP and all ‘other’ methods (including condoms and no method). P-values reported from the generalized logit regression indicate that the choice of contraceptive method was significantly related to the exposure factor in question without giving information about where these differences lie. A comparative regression analysis was also performed to examine method choice by ethnic group to identify any factors that were associated with a greater likelihood of choosing a LARC method.

## Results

### Characteristics of participants

We identified 320 Chinese women from the electronic records pertaining to women presenting to the hospital abortion clinic over the 43 month period covering August 2006 to February 2010. Fifteen women were excluded for reasons that included: missing records (2), incorrectly identified as Chinese (11), abortion sought for foetal anomaly (2). Women who had more than one abortion during the data collection period were included only once (the most recent procedure was included).

Table 1 presents the demographic and clinical characteristics of Chinese women presenting for abortion during the data collection period, with comparative data for European and Maori women. The mean age of Chinese women was 27.6 years old (range 16–48 years, SD 6.5), which was significantly older (by three years) than women in comparison groups. European women had a mean age of 24.2 years (range 14–43 years, SD 6.8) and Maori women a mean age of 24.9 years (range 14–41 years, SD 6.6), p<0.05. Chinese and European women presented at an earlier gestational age (mean 8.5 and 8.6 weeks respectively) than Maori women (10 weeks), p<0.05 and were significantly more likely to be presenting with a first pregnancy than Maori women (p<0.05). The proportion of Chinese women who had one or more previous abortions (41%) was significantly higher than for European women (32%) p<0.05, but did not differ significantly from Maori women (49.2%), p>0.05. Chinese women were significantly less likely to have children than women in comparison groups (p<0.05), were less likely to be smokers (p<0.05) and had a significantly lower rate of Chlamydia infection than both European and Maori women (p<0.05).

**Table 1:** Demographic and clinical characteristics of women presenting for abortion at a public hospital clinic.

Characteristic	Chinese		European		Maori		Chi square p-value
	(n = 305)		(n = 277)		(n = 128)		
	n	%	n	%	n	%	
Age-band							
19 years and younger	15	4.9	90	32.5	27	21.1	<0.05
20 to 24 years	104	34.1	82	29.6	45	35.2	
25 to 29 years	96	31.5	43	15.5	24	18.8	
30 to 34 years	32	10.5	31	11.2	20	15.6	
35+ years	59	19.3	31	11.2	12	9.4	
Socioeconomic status							
Least deprived (1–3)	76	24.9	56	20.2	16	12.5	<0.05
Moderately deprived (4–7)	110	36.1	106	38.3	33	25.8	
Most deprived (8–10)	119	39.0	112	40.4	79	61.7	
Not known	0	0	3	1.1	0	0.0	
Procedure							
Surgical	289	94.8	248	89.5	123	96.1	ns
Medical	16	5.2	29	10.5	5	3.9	
Gestational age							
1^st^ trimester (≤13 weeks)	294	96.4	265	95.7	120	93.8	ns
2^nd^ trimester (14+ weeks)	11	3.6	12	4.3	8	6.3	
Gravida							
One	138	45.2	122	44.0	22	17.2	<0.05
Two	69	22.6	50	18.1	26	20.3	
Three or more	98	32.1	105	37.9	80	62.5	
Previous abortion							
None	180	59.0	188	67.9	65	50.8	<0.05
One	95	31.1	62	22.4	32	25.0	
Two or more	30	9.8	27	9.7	31	24.2	
Parity							
Nulliparous	215	70.5	162	58.5	38	29.7	<0.05
Primiparous	30	9.8	48	17.3	33	25.8	
Multiparous	60	19.7	67	24.2	57	44.5	
Smoker							
Yes	25	8.2	118	42.6	75	58.6	<0.05
No	265	86.9	129	46.6	41	32.0	
Not known	15	4.9	30	10.8	12	9.4	
Sexually transmitted infections							
Chlamydia (positive)	14	4.6	22	7.9	16	12.5	<0.05
Result unknown	5	1.6	8	2.9	4	3.1	

The majority of Chinese women were overseas-born (93%, 284/305), with the highest proportion from mainland China (86%, 262/305). A small number of women emigrated from Malaysia, Hong Kong, Taiwan or other Asian countries (7.2%, 22/305), 6.6% were New Zealand-born (20/305) and country of origin was not known for one woman. Over half the sample had obtained residency status in New Zealand (55%, 168/305), and of those who were not residents, 51% (70/137) held a student visa, and 42% (57/137) a work visa. Data on length of stay showed that 9.2% of overseas-born women were recent arrivals having been in New Zealand for less than two years (26/284), 38.9% (111/305) had resided in New Zealand for 2–5 years and 29.6% (84/284) for 6–10 years. A third of the women (34%, 104/305) were seen at the clinic with an interpreter (although this may be an underestimate as this information was not recorded for 79 patients).

### Contraceptive method use pre-abortion

Table 2 presents methods of contraception reportedly in use pre-abortion and methods chosen for intended use post-abortion. Just over half the women had used no method of contraception pre-abortion, with no significant difference between the three ethnic groups. Chinese women who had been in New Zealand for less than two years had the highest non-use of contraception (73%), compared with women who had been in New Zealand for 2–5 years (49%), for 6–10 years (58%) or for longer than 10 years (44%). New Zealand born Chinese women had the lowest non-use of contraception (40%), however an overall chi-square test for significance showed that this difference was not statistically significant (p = 0.09).

**Table 2:** Peri-abortion contraceptive choices of Chinese, European and Maori women seeking abortion at a public hospital clinic.

Method of Contraception	Chinese			European			Maori		
	(n = 305)			(n = 277)			(n = 128)		
	n	%	(95% CI)	n	%	(95% CI)	n	%	(95% CI)
Pre-abortion									
No method	167	54.8	(49–60.4)	141	50.9	(44.9–56.9)	75	58.6	(49.6–67.2)
Condoms	78	25.6	(20.8–30.9)	60	21.7	(17.0–27.0)	23	18.0	(11.7–25.7)
OCP	11	3.6	(1.8–6.4)*	46	16.6	(12.4–21.5)	17	13.3	(7.9–20.4)
Emergency pill	12	3.9	(2.0–6.8)	14	5.1	(2.8–8.3)	0	0	(0–2.3)
DMPA	1	0.3	(0–1.8)	0	0	(0–1.1)	0	0	(0–2.3)
IUD	6	2.0	(0.7–4.2)	2	0.7	(0.1–2.6)	0	0	(0–2.3)
Natural family planning^a^	18	5.9	(3.5–9.2)*	3	1.1	(0.2–3.1)	2	1.6	(0.2–5.5)
Withdrawal	9	3.3	(1.6–5.9)*	0	0	(0–1.1)	1	0.8	(0–4.3)
Diaphragm	1	0.3	(0–1.8)	0	0	(0–1.1)	0	0	(0–2.3)
Not recorded	2	0.7	(0.1–2.3)	11	4.0	(2.0–7.0)	10	7.8	(3.8–13.9)
Post-abortion									
Condoms	32	10.5	(7.3–14.5)*	11	4.0	(2.0–7.0)	0	0	(0.0–2.3)
OCP	143	46.9	(41–52.7)*	121	43.7	(37.8–49.7)	32	25.0	(17.8–33.4)
DMPA	14	4.6	(2.5–7.6)*	40	14.4	(10.5–19.1)	34	26.6	(19.1–35.1)
Mirena LNG-IUS®	25	8.2	(5.4–11.9)	15	5.4	(3.1–8.8)	12	9.4	(4.9–15.8)
Multiload copper IUD	63	20.7	(16.3–25.6)	45	16.2	(12.1–21.1)	35	27.3	(19.8–35.9)
Total IUD use	88	28.9	(23.8–34.3)*	60	21.7	(17.0–27.0)	47	36.7	(28.4–45.7)
Other^b^	28	9.2	(6.2–13.0)	45	16.2	(12.1–21.1)	15	11.7	(6.7–18.6)

a Natural family planning refers to the rhythm method or fertility awareness.

b Other methods chosen by Chinese women post-abortion include: abstinence (2), partner to have vasectomy (1), none/undecided/wishes to discuss with own doctor (25).

* Denotes a significant difference in method use by Chinese women and one or both comparison groups based on confidence intervals.

Chinese women were more likely to report having used condoms (25.6%) than Maori women (18%), and were significantly less likely than both European and Maori women to have been using the OCP. A significantly greater proportion of Chinese women (5.9%) were using ‘natural’ family planning or fertility awareness based methods than women in comparison groups (1.1% of European and 1.6% of Maori women). Numbers of Chinese women using different contraceptive methods pre-abortion were too small to perform analyses in relation to length of residency in New Zealand.

### Contraceptive method choice post-abortion

The OCP was chosen for intended use post-abortion by 46.9% of Chinese women and 43.7% of European women, proportions that were significantly higher than for Maori women (25%). Intent to use condoms as their primary form of contraception post-abortion was higher among Chinese women (10.5%) than European (4%) and Maori women (0%). Fewer Chinese women chose DMPA (4.6%) than either European (14.4%) or Maori women (25%). Use of an IUD (either a copper multiload or Mirena LNG-IUS) was highest among Maori women (36.7%), followed by Chinese (28.9%) then European women (21.7%). The difference between Chinese and Maori was not significant, but both these groups had marginally higher uptake of the IUD than European women. No difference was observed between ethnic groups in the proportions of women choosing a hormone versus a non-hormone containing IUD.

**Figure 1:**
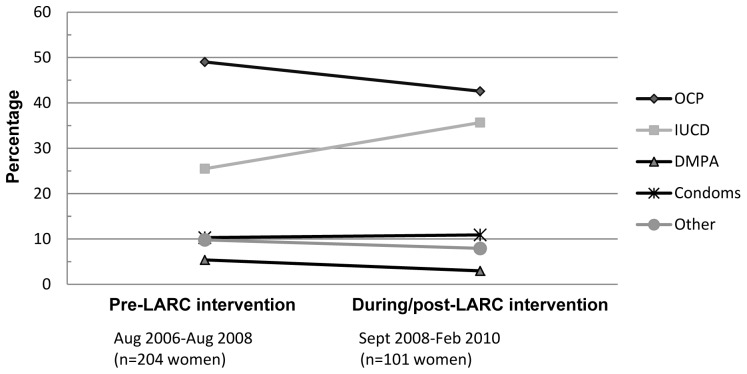
Post-abortion contraceptive method use by Chinese women prior to and after an intervention study at the clinic that aimed to improve use of LARC methods.

Due to the differences in data collection periods for the comparison groups, and a move towards promoting the benefits of LARC methods (particularly IUDs) to women attending this clinic from late 2008 onwards, we examined post-abortion method choice for Chinese women over time (Figure 1). Around half of women (49%) were choosing the OCP between Aug 2006 and Aug 2008 (pre-LARC intervention), but this dropped slightly to 42.6% during Sept 2008 to Feb 2010 (post-LARC intervention), with a corresponding increase in choice of an IUD. The small number of Chinese women seen at the clinic during the intervention study (n = 10) are included in the post-intervention group, of whom 2 chose an IUD, and 8 the OCP.

### Correlates of methods chosen by Chinese women

Table 3 presents the results of logistic regression analyses (with odds ratios and 95% confidence intervals) that were used to identify factors associated with post-abortion method choice by Chinese women. Odds ratios (OR) greater than 1 indicate whether a particular factor is associated with a greater likelihood of choosing a particular category of method relative to the reference method category. Three factors were significantly related to method choice: age, parity and previous abortion (p<0.05). Women in older age-bands (both 25–34 and 35+) were more likely to choose LARC methods over the OCP than women aged 24 years or younger (OR 2.5, 95% CI 1.14 to 5.46 for age 25–34, and OR 6.03, 95% CI 1.34–27.09 for age 35+, relative to age 24 or under). The relative likelihood of choosing the OCP declined with increasing age (OR 0.12, 95% CI 0.03–0.61 for age 35+). Parity was strongly related to method choice: women with children were more likely to choose LARC methods over the OCP than nulliparous women (OR 10.1, 95% CI 2.42–42.19 for primiparous women, OR 15.14, 95% CI 4.33–52.85 for multiparous women). Previous abortion was also associated with method choice: women who had two or more previous abortions were more likely to choose a LARC method over an OCP than women presenting for their first abortion (OR 5.52, 95% CI 1.69–17.99). Time spent in New Zealand, socioeconomic deprivation and smoking status were not significantly related to method choice (p>0.05 for all factors).

**Table 3:** Logistic regression for likelihood of Chinese women choosing a LARC, OCP or Other method according to demographic and pregnancy history.

Factor	LARC vs OCP		OCP vs Other^a b^		Factor p-value
	OR	(95% CI)	OR	(95% CI)	
Age group					
24 or under	*Reference*		*Reference*		0.026
25 to 34	2.5	(1.14–5.46)	0.63	(0.3–1.34)	
35+	6.03	(1.34–27.09)	0.12	(0.03–0.61)	
Time in New Zealand (NZ)					
NZ-born (n = 20)	*Reference*		*Reference*		0.144
10+ years (n = 36)	0.8	(0.14–4.58)	0.17	(0.02–1.97)	
6 to 10 years (n = 84)	0.61	(0.15–2.54)	0.25	(0.03–2.28)	
2 to 5 years (n = 111)	0.85	(0.21–3.44)	0.29	(0.03–2.59)	
Less than 2 years (n = 26)	1.78	(0.35–8.95)	0.28	(0.03–3.13)	
Not known (n = 28)	0.19	(0.03–1.29)	0.13	(0.01–1.33)	
Socioeconomic status					
Least deprived (1–3)	*Reference*		*Reference*		0.978
Moderately deprived (4–7)	1.24	(0.53–2.95)	1.02	(0.42–2.47)	
Most deprived (8–10)	1.07	(0.46–2.47)	0.93	(0.4–2.2)	
Parity					
Nulliparous	*Reference*		*Reference*		<0.001
Primiparous	10.1	(2.42–42.19)	0.3	(0.06–1.47)	
Multiparous	15.14	(4.33–52.85)	0.44	(0.1–1.86)	
Previous abortion					
None	*Reference*		*Reference*		0.003
One	0.89	(0.43–1.86)	1.46	(0.69–3.1)	
Two or more	5.52	(1.69–17.99)	6.8	(0.67–66.7)	
Smoker					
No	*Reference*		*Reference*		0.161
Yes	2.22	(0.67–7.32)	0.95	(0.24–3.86)	
Don't know	5.35	(1.09–26.18)	0.22	(0.05–1.02)	

a Other methods here include: condoms (n = 32), going to discuss with own Doctor, none, withdrawal or abstinence (n = 28).

b Due to the heterogeneous nature of the ‘Other’ methods category, the odds ratios for the Other vs OCP comparison have been reversed for ease of interpretation. That is, the odds ratios presented here describe the likelihood of choosing the OCP rather than ‘Other’ methods (the odds ratios presented for OCP vs Other are the direct inverse of those for Other vs OCP)

### Correlates of method choice across ethnic groups

An initial logistic regression model combining data for all three groups suggested that the relationship between method choice and parity/previous abortion differed by ethnicity (significant interactions in the overall model were ethnicity by parity p = 0.006, ethnicity by previous abortion p = 0.03). The analysis was then run separately for each ethnic group including the following predictor variables: deprivation, age group, parity and previous abortion. For both European and Maori women, parity was the only factor significantly related to choice of method (p<0.05) controlling for deprivation, age and previous abortion. That is, women with more children were more likely to choose a LARC method. The strength of association between parity and method choice differed between groups with a stronger association observed for Chinese women (for those with two or more children OR 8.95, 95% CI 3.52–22.7), followed by European women (OR 5.21, 95% CI 2.38–11.4), then Maori (OR 3.98, 95% CI 1.41–11.3).

### Clinic attendance by ethnic groups (in relation to population data)

Nine percent of women presenting for abortion at this clinic during the data collection period were of Asian ethnicities (871/9513) and 3.4% were Chinese (320/9513). These percentages closely matched the population percentages of reproductive-aged women in these ethnic groups in the study area. 2006 New Zealand regional census data showed that Asian women aged 15–44 years made up 10.5% of the population (10,098/95,910) [3] and Chinese women made up 4.7% of the population (4461/95,910) in the area served by the clinic. [14] By comparison, 49% of women presenting for abortion (during 2006–2009) were European, a group who made up 64.3% of the region's population aged 15–44 years. Maori women made up 25% of women presenting for an abortion but only 12.9% of the population. [3]

## Discussion

This analysis of contraceptive use by women seeking abortion showed that around half of all clinic attendees used no method of contraception around the time of conception, regardless of their ethnicity. Use of the OCP was low among Chinese women prior to their clinic visit, a quarter had been using condoms, and small numbers had been using behavioral family planning methods. Following contraceptive counseling, intended use of the OCP was similar to that of European women (and higher than for Maori). Close to a third of Chinese women chose an IUD but few women chose DMPA for post-abortion use. Intended use of condoms as a primary method of post-abortion contraception was higher among Chinese women than both comparison groups.

Pre-abortion method use by Chinese women in this study was consistent with use reported in two qualitative studies involving Chinese women presenting for abortion in Canada, [8], [10] and with method use reported elsewhere in New Zealand. [5] By contrast, post-abortion method choices differ to those described in the earlier New Zealand study in which Chinese women were reported to have a ‘profound reluctance to try any form of contraception other than a condom, and seldom consider using oral contraceptives.’ [5] The OCP was chosen for use by 47% of all Chinese women. Younger Chinese women were more likely to select this method, with 68% of under 25 year olds choosing the OCP. Chinese women had good uptake of the IUD, similar to the rate of uptake by Maori women, and marginally higher than European women.

Parity was the most significant predictor of choice of long-acting method; parous women had a higher uptake of the IUD than non-parous women. This probably reflects the commonly held view that IUDs are most appropriately prescribed to women who have had children. Women who had two or more prior abortions also had a higher uptake of LARC methods, so may have been more motivated to avoid subsequent unwanted pregnancies. An increase in IUD use by Chinese women (with a corresponding drop in choice of the OCP) was observed in 2009–2010, this change is likely to reflect the impact of an intervention study that was run in this clinic in September 2008 to promote uptake of LARC methods. [13]

Length of stay in New Zealand had no effect on choice of method for use post-abortion in the present study, but we observed a higher (but not statistically significant) proportion of new arrivals using no method pre-abortion than women who had been in New Zealand for a longer period of time. This finding differs to that of a study of Asian immigrants in the United States in which use of the OCP increased corresponding to length of time since migration. [16] The number of women reviewed in the present study might not have been adequate to observe significant differences in post-abortion method choice by length of stay. Furthermore, our sample includes only those women seeking an abortion who are less likely to be using effective contraceptive methods than women in the general population, so it is difficult to draw conclusions about method use in relation to length of stay.

The majority of Chinese women seeking abortion at this clinic were overseas-born; close to half had been in New Zealand for less than five years and just over half were permanent residents. By comparison, national data show that 78% of the Asian population are overseas-born. [3] Interestingly, Chinese women were significantly older, and less likely to have had a Chlamydia diagnosis than other groups. By describing the ethnicity of clinic attendees as a proportion of the reproductive aged female population in the clinic catchment area, we have shown that Asian women were not over-represented among women seeking abortion in this area. While previously reported abortion ratios (rate per 1000 known pregnancies) suggest that Asian women are more likely to terminate than to proceed with a pregnancy, reporting the data in this way gives no indication of the population rate of pregnancies among reproductive aged women by ethnic group, nor details of planned versus unplanned pregnancies. Fertility rates (live births per 1000 resident female population) are in fact lowest for Asian women, [17] so of the comparatively lower numbers of Asian women getting pregnant, a greater number are choosing to terminate the pregnancy. Together with the finding that Chinese women had the same rate of contraceptive ‘non-use’ prior to the abortion, we would not conclude that Chinese women surveyed here were using abortion as a form of contraception as has been reported in the media. [1]

Access to healthcare is one of the many challenges faced by migrant communities when settling in a new country. A 2005 survey on access to health and social services by Chinese in Auckland, New Zealand reported that a lack of English language proficiency, communication difficulties and a lack of awareness about service availability and the role of primary health care and General Practitioners (family physicians) as a first point of contact were key issues. [18] Asian people are reported to be less likely to access primary care in New Zealand, [19], [20] and Asian women less likely to see a doctor about contraception than other ethnic groups. [20] The likelihood of having a usual GP (family physician) is lower among those who have been in the country for fewer than five years than among those who were born in New Zealand or have lived there for more than 10 years. [21] It is perhaps not surprising that Chinese migrants in New Zealand have a lower use of contraceptive methods requiring a GP visit and prescription, so rely on condoms and behavioral methods of contraception. This hypothesis would ideally be tested in further research.

Although post-abortion uptake of both the OCP and IUD by Chinese women in the present study was high, rates of contraceptive method discontinuation are also known to be high thereby increasing individuals risk of unplanned pregnancy. [22] Analysis of data collected in a large US survey revealed overall method discontinuance of 68% at 12 months, with around 31% of OCP users stopping by 6 months and 47% stopping within a year. [22] IUD removals in a New Zealand study involving women who choose an IUD for immediate post-abortion insertion were lower, with 20% removed at 6 months, primarily due to side effects. [13] A small observational study in Canada reported that only 66% of East Asian immigrants used any of the OCP samples provided for post-abortion use in the first month, and this had dropped to 47% by the second month. [23] There is some evidence to suggest that Asian and Caucasian women have a different physiological response to hormonal contraceptives that influences their experience of side effects. [23], [24] However, in the Canadian study, East Asian women were less likely to start, and more likely to stop hormonal contraceptives than the Caucasian Canadian women for reasons other than actual side effects. [23]

### Study limitations

A limitation of this study was that data were collected from only one clinic, and from women seeking an abortion, so the choices made by overseas-born Chinese women in the present study may not be generalized beyond the study participants. However the clinic is the second largest abortion provider in the country and is the sole provider in a large geographic region. Comparative data for European and Maori women were collected during only one year (2008) over a 10-week period. Data for Chinese women were collected over a three and a half year period (Aug 2006–Feb 2010) to obtain a large enough sample for the comparative analysis, and included patients seen both before and after a 10-week intervention study that aimed to increase awareness and uptake of LARC methods for post-abortion use. Furthermore, key differences were observed between ethnic groups in terms of age, socioeconomic deprivation and previous pregnancy history that might have contributed to differences observed between groups. However, regression analyses were designed to control for these potential confounding variables. Attitudes towards contraceptive methods and reasons for method choice were not sought in the present study, but would be helpful to explain the differences we observed.

The high proportion of women choosing hormonal methods or the copper-IUD following counseling may not reflect the likely uptake of these methods by women in the general Chinese community, even if they were to receive contraceptive counseling and information. Women in the present study were presenting with an unwanted pregnancy so are likely to have been more motivated to choose a more effective method than they had previously used. DMPA is considered a LARC method in New Zealand and in the United Kingdom [7] (so was grouped accordingly in the regression analysis), but its inclusion in this category can be debated given the significantly shorter period of protection against pregnancy compared with intrauterine and implantable methods. LARC methods are inserted or administered at the clinic prior to discharge whereas oral contraceptives are prescribed, but follow-up is not undertaken to determine whether prescriptions are filled, therefore actual uptake of the OCP was unknown.

### Conclusions

This study provides evidence that is contrary to the negative media reports on the sexual health of Chinese migrant women in New Zealand that have been highlighted previously. [1] Chinese women had the same likelihood of contraceptive non-use pre-abortion as women of other ethnicities, with a greater preference for use of fertility awareness based methods for pregnancy prevention. The present study showed that when given contraceptive counseling with information on risks, benefits and side effects of a range of methods, Chinese women were willing to choose effective hormone-containing methods of contraception for use following an abortion. This suggests that women's concerns about side effects (such as weight gain and infertility), [8] might be allayed with the provision of adequate information. We suggest there is an unmet need for provision of contraceptive information to migrant Chinese women in New Zealand. With increasing numbers of reproductive aged Chinese migrants expected in New Zealand over the next ten to fifteen years,[2_ENREF_2] it is important that strategies are implemented to ensure women are educated about, and aware of contraceptive options before they face an unwanted pregnancy.
